# Rate of convergence by Kantorovich-Szász type operators based on Brenke type polynomials

**DOI:** 10.1186/s13660-017-1430-z

**Published:** 2017-06-29

**Authors:** Tarul Garg, Purshottam Narain Agrawal, Serkan Araci

**Affiliations:** 1 Department of Mathematics, Indian Institute of Technology Roorkee, Roorkee, 247667 India; 2 Department of Economics, Faculty of Economics, Administrative and Social Sciences, Hasan Kalyoncu University, Gaziantep, 27410 Turkey

**Keywords:** 41A10, 41A25, 41A36, Brenke type polynomials, Szász operator, Ditzian-Totik modulus of smoothness, derivative of bounded variation, Peetre’s K-functional, rate of convergence

## Abstract

The present paper deals with the approximation properties of the univariate operators which are the generalization of the Kantorovich-Szász type operators involving Brenke type polynomials. We investigate the order of convergence by using Peetre’s K-functional and the Ditzian-Totik modulus of smoothness and study the degree of approximation of the univariate operators for continuous functions in a Lipschitz space, a Lipschitz type maximal function and a weighted space. The rate of approximation of functions having derivatives equivalent with a function of bounded variation is also obtained.

## Introduction

Linear positive operators play an important role in the study of approximation theory. One of the best known among these operators is the Szász operator [1]

$$ S_{n}(f;x)= e^{-nx} \sum_{k=0}^{\infty} \frac{(n x)^{k}}{k!} f \biggl(\frac {k}{n} \biggr), \quad x\geq0, n\in \mathbb{N}, $$

a generalization of Bernstein polynomials to the infinite interval. Jakimovski and Leviatan [2] generalized Szász operators by means of the Appell polynomials. Subsequently, Ismail [3] gave another generalization of the Szász operators by involving the Sheffer polynomials. Varma et al. [4] presented yet another generalization of the Szász operators by utilizing the Brenke type polynomials and showed that these polynomials include the Appell polynomials and Gould-Hopper polynomials as special cases. Varma and Taşdelen [5] determined a link between the Szász operators and the discrete orthogonal polynomials, e.g., Charlier polynomials, and established the Korovkin type theorem and the rate of convergence by means of the classical modulus of continuity. Taşdelen et al. [6] introduced a Kantorovich variant of the Szász operators based on Brenke type polynomials and discussed the order of convergence with the aid of the moduli of continuity and Peetre’s K-functional. Öksüzer et al. [7] estimated the rate of convergence for functions of bounded variation for these operators by means of some results of probability theory. Later on, Aktaş et al. [8] considered a Stancu type modification of the Szász Kantorovich operators involving Brenke type polynomials and obtained the degree of approximation by means of the classical modulus of continuity and Peetre’s K-functional. A Voronovskaya type theorem for the considered operators including Gould-Hopper polynomials was also proven. Mursaleen and Ansari [9] presented a Chlodowsky type generalization of Szász operators defined by using Brenke type polynomials and studied the order of convergence for functions in a weighted space besides other classical approximation results.

Recently, for $f \in C_{E}[0,\infty):= \{f\in C[0,\infty): |f(t)| \leq a e^{bt}, a\in\mathbb{R}^{+}, b\in\mathbb{R}\}$, Atakut and Büyükyazici [10] considered the Kantorovich-Szász type operators involving Brenke type polynomials as

1 $$ L_{n}^{\alpha_{n}, \beta_{n}}(f;x) = \frac{\beta_{n}}{A(1) B(\alpha_{n} x)} \sum _{k=0}^{\infty} p_{k}( \alpha_{n} x) \int_{k/\beta_{n}}^{(k+1)/\beta_{n}} f(t) \,dt, $$

where $\{ \alpha_{n}\}$, $\{ \beta_{n}\}$ are strictly increasing sequences of positive numbers such that

2 $$ \lim_{n\rightarrow\infty} \frac{1}{\beta_{n}} = 0,\qquad \frac{\alpha_{n}}{\beta _{n}}= 1+O \biggl( \frac{1}{\beta_{n}} \biggr),\quad \mbox{as } n \rightarrow\infty $$

and $p_{k}(x)=\sum_{r=0}^{\infty} a_{k-r}b_{r} x^{r}$, $k=0,1,2,\ldots$ , are Brenke type polynomials having the following generating function:

3 $$ A(t) B(xt)=\sum_{k=0}^{\infty}p_{k}(x)t^{k}, $$

where $A(t)$, $B(t)$ are analytic functions such that

4 $$ \begin{aligned} &A(t)=\sum_{r=0}^{\infty}a_{r} t^{r}, \quad a_{0}\neq0, \\ &B(t)=\sum_{r=0}^{\infty}b_{r} t^{r},\quad b_{r}\neq0\ (r\geq0) \end{aligned} $$

hold. Assume that

(i) $A(1)\neq0$, $\frac{a_{k-r}b_{r}}{A(1)}\geq0$ for $0\leq r \leq k$, $k=0,1,2,\ldots$ ,
(ii) $B:[0,\infty) \rightarrow(0,\infty)$,
(iii) (3) and the power series (4) converges for $|t|< R$ ($R>1$),
(iv) $\lim_{y \rightarrow\infty} \frac{B^{(k)}(y)}{B(y)}= 1$ for $k=1,2,3,4$.

Atakut and Büyükyazici [10] studied the order of convergence of the operators defined in (1). For the special case $\alpha_{n} = \beta_{n} = n$, the operators (1) are reduced to the Kantorovich variant of a generalization of Szász operators based on the Brenke type polynomials. For some other significant papers dealing with the generalization of Szász type operators, we refer to [11–13].

In the present paper we establish the order of approximation of the operators (1) by using Peetre’s K-functional and the Ditzian-Totik modulus of smoothness. Also, we study the rate of convergence of these operators for a Lipschitz space, a Lipschitz type maximal function and a weighted space. The rate of approximation of functions having derivatives equivalent to a function of bounded variation is also obtained.

## Preliminaries

To examine the approximation properties of the operator $L_{n}^{\alpha_{n}, \beta_{n}}$ defined in (1), we give some basic results in the form of lemmas as follows.

### Lemma 2.1

[10]

*For* $x\in[0,\infty )$, *we have*

$$\begin{aligned} (\mathrm{i})&\quad L_{n}^{\alpha_{n}, \beta_{n}}(1;x)= 1 , \\ (\mathrm{ii})&\quad L_{n}^{\alpha_{n}, \beta_{n}}(t;x)= \frac{\alpha_{n}}{\beta_{n}} \frac{B'(\alpha_{n} x)}{B(\alpha_{n} x)} x + \frac{1}{\beta_{n}} \frac {2A'(1)+A(1)}{2A(1)} , \\ (\mathrm{iii})&\quad L_{n}^{\alpha_{n}, \beta_{n}}\bigl(t^{2};x \bigr)= \frac{\alpha_{n}^{2}}{\beta _{n}^{2}} \frac{B''(\alpha_{n} x)}{B(\alpha_{n} x)} x^{2} + \frac{2\alpha_{n}}{\beta_{n}^{2}} \frac{A'(1)+A(1)}{A(1)} \frac{B'(\alpha_{n} x)}{B(\alpha_{n} x)} x \\ &\quad \hphantom{L_{n}^{\alpha_{n}, \beta_{n}}\bigl(t^{2};x \bigr)={}}{}+ \frac {1}{3\beta_{n}^{2}} \frac{3A''(1)+6A'(1)+A(1)}{A(1)} , \\ (\mathrm{iv})&\quad L_{n}^{\alpha_{n}, \beta_{n}}\bigl(t^{3};x \bigr)= \frac{\alpha_{n}^{3}}{\beta _{n}^{3}} \frac{B'''(\alpha_{n} x)}{B(\alpha_{n} x)} x^{3} + \frac{3\alpha _{n}^{2}}{2\beta_{n}^{3}} \frac{2A'(1)+3A(1)}{A(1)} \frac{B''(\alpha_{n} x)}{B(\alpha_{n} x)} x^{2} \\ &\quad\hphantom{L_{n}^{\alpha_{n}, \beta_{n}}\bigl(t^{3};x \bigr)={}}{}+ \frac{\alpha_{n}}{2\beta_{n}^{3}} \frac{6A''(1)+18A'(1)+7A(1)}{A(1)} \frac{B'(\alpha_{n} x)}{B(\alpha_{n} x)} x \\ &\quad\hphantom{L_{n}^{\alpha_{n}, \beta_{n}}\bigl(t^{3};x \bigr)={}}{}+ \frac{1}{4\beta_{n}^{3}} \frac {4A'''(1)+18A''(1)+14A'(1)+A(1)}{A(1)} , \\ (\mathrm{v})&\quad L_{n}^{\alpha_{n}, \beta_{n}}\bigl(t^{4};x \bigr)= \frac{\alpha_{n}^{4}}{\beta _{n}^{4}} \frac{B^{(4)}(\alpha_{n} x)}{B(\alpha_{n} x)} x^{4} + \frac{4\alpha _{n}^{3}}{\beta_{n}^{4}} \frac{A'(1)+2A(1)}{A(1)} \frac{B'''(\alpha_{n} x)}{B(\alpha_{n} x)} x^{3} \\ &\quad \hphantom{L_{n}^{\alpha_{n}, \beta_{n}}\bigl(t^{3};x \bigr)={}}{}+ \frac{3\alpha_{n}^{2}}{\beta_{n}^{4}} \frac{2A''(1)+8A'(1)+5A(1)}{A(1)} \frac{B''(\alpha_{n} x)}{B(\alpha_{n} x)} x^{2} \\ &\quad \hphantom{L_{n}^{\alpha_{n}, \beta_{n}}\bigl(t^{3};x \bigr)={}}{}+ \frac{\alpha_{n}}{\beta_{n}^{4}} \frac{4A'''(1)+24A''(1)+30A'(1)+6A(1)}{A(1)} \frac{B'(\alpha_{n} x)}{B(\alpha_{n} x)} x \\ &\quad \hphantom{L_{n}^{\alpha_{n}, \beta_{n}}\bigl(t^{3};x \bigr)={}}{}+ \frac{1}{5\beta_{n}^{4}} \frac {5A^{(4)}(1)+40A'''(1)+75A''(1)+30A'(1)+A(1)}{A(1)} . \end{aligned}$$

As a consequence of Lemma 2.1, we have the following.

### Lemma 2.2

*For* $x\in[0,\infty)$,

$$\begin{aligned} (\mathrm{i})&\quad L_{n}^{\alpha_{n}, \beta_{n}}(t-x;x) = \biggl( \frac{\alpha _{n}}{\beta_{n}} \frac{B'(\alpha_{n} x)}{B(\alpha_{n} x)}-1 \biggr) x + \frac {1}{\beta_{n}} \frac{2A'(1)+A(1)}{2A(1)} , \\ (\mathrm{ii})&\quad L_{n}^{\alpha_{n}, \beta_{n}}\bigl((t-x)^{2};x \bigr) = \biggl( \frac{\alpha _{n}^{2}}{\beta_{n}^{2}} \frac{B''(\alpha_{n} x)}{B(\alpha_{n} x)} - \frac{2\alpha _{n}}{\beta_{n}} \frac{B'(\alpha_{n} x)}{B(\alpha_{n} x)}+1 \biggr) x^{2} \\ &\quad \hphantom{L_{n}^{\alpha_{n}, \beta_{n}}\bigl((t-x)^{2};x \bigr) ={}}{}+ \biggl( \frac {2\alpha_{n}}{\beta_{n}^{2}} \frac{A'(1)+A(1)}{A(1)} \frac {B'(\alpha_{n} x)}{B(\alpha_{n} x)} - \frac{1}{\beta_{n}} \frac{2A'(1)+A(1)}{2A(1)} \biggr) x \\ &\quad \hphantom{L_{n}^{\alpha_{n}, \beta_{n}}\bigl((t-x)^{2};x \bigr) ={}}{}+ \frac{1}{3\beta_{n}^{2}} \frac{3A''(1)+6A'(1)+A(1)}{A(1)} , \\ (\mathrm{iii})&\quad L_{n}^{\alpha_{n}, \beta_{n}}\bigl((t-x)^{4};x \bigr) = \biggl( \frac{\alpha _{n}^{4}}{\beta_{n}^{4}} \frac{B^{(4)}(\alpha_{n} x)}{B(\alpha_{n} x)} - \frac{4\alpha_{n}^{3}}{\beta_{n}^{3}} \frac{B'''(\alpha_{n} x)}{B(\alpha_{n} x)} + \frac{6\alpha_{n}^{2}}{\beta_{n}^{2}} \frac{B''(\alpha_{n} x)}{B(\alpha_{n} x)} \\ &\quad \hphantom{L_{n}^{\alpha_{n}, \beta_{n}}\bigl((t-x)^{4};x \bigr) ={}}{}- \frac{4\alpha_{n}}{\beta_{n}} \frac{B'(\alpha_{n} x)}{B(\alpha_{n} x)} + 1 \biggr) x^{4} + \biggl( \frac{4\alpha_{n}^{3}}{\beta_{n}^{4}} \frac{A'(1)+2A(1)}{A(1)} \frac{B'''(\alpha_{n} x)}{B(\alpha_{n} x)} \\ &\quad \hphantom{L_{n}^{\alpha_{n}, \beta_{n}}\bigl((t-x)^{4};x \bigr) ={}}{}- \frac {6\alpha_{n}^{2}}{\beta_{n}^{3}} \frac{2A'(1)+3A(1)}{A(1)} \frac{B''(\alpha_{n} x)}{B(\alpha_{n} x)} + \frac{12\alpha_{n}}{\beta_{n}^{2}} \frac {A'(1)+A(1)}{A(1)} \frac{B'(\alpha_{n} x)}{B(\alpha_{n} x)} \\ &\quad \hphantom{L_{n}^{\alpha_{n}, \beta_{n}}\bigl((t-x)^{4};x \bigr) ={}}{}-\frac{2}{\beta_{n}} \frac{2A'(1)+A(1)}{A(1)} \biggr) x^{3} + \biggl(\frac{3\alpha_{n}^{2}}{\beta _{n}^{4}} \frac{2A''(1)+8A'(1)+5A(1)}{A(1)} \\ &\quad \hphantom{L_{n}^{\alpha_{n}, \beta_{n}}\bigl((t-x)^{4};x \bigr) ={}}{}\times\frac{B''(\alpha_{n} x)}{B(\alpha _{n} x)} - \frac{2\alpha_{n}}{\beta_{n}^{3}} \frac {6A''(1)+18A'(1)+7A(1)}{A(1)} \frac{B'(\alpha_{n} x)}{B(\alpha_{n} x)} \\ &\quad \hphantom{L_{n}^{\alpha_{n}, \beta_{n}}\bigl((t-x)^{4};x \bigr) ={}}{} + \frac{2}{\beta_{n}^{2}} \frac{3A''(1)+6A'(1)+A(1)}{A(1)} \biggr) x^{2} \\ &\quad \hphantom{L_{n}^{\alpha_{n}, \beta_{n}}\bigl((t-x)^{4};x \bigr) ={}}{}+ \biggl( \frac{\alpha _{n}}{\beta_{n}^{4}} \frac{4A'''(1)+24A''(1)+30A'(1)+6A(1)}{A(1)} \frac {B'(\alpha_{n} x)}{B(\alpha_{n} x)} \\ &\quad \hphantom{L_{n}^{\alpha_{n}, \beta_{n}}\bigl((t-x)^{4};x \bigr) ={}}{}- \frac{1}{\beta_{n}^{3}} \frac {4A'''(1)+18A''(1)+14A'(1)+A(1)}{A(1)} \biggr) x \\ &\quad \hphantom{L_{n}^{\alpha_{n}, \beta_{n}}\bigl((t-x)^{4};x \bigr) ={}}{}+ \frac{1}{5\beta_{n}^{4}} \frac{5A^{(4)}(1)+40A'''(1)+75A''(1)+30A'(1)+A(1)}{A(1)} . \end{aligned}$$

## Local theorem of approximation

In what follows, let $L_{n}^{\alpha_{n}, \beta_{n}}(t-x;x) = \gamma_{\alpha _{n},\beta_{n}}(x)$ and $L_{n}^{\alpha_{n}, \beta_{n}}((t-x)^{2};x)= \delta^{2}_{\alpha_{n},\beta_{n}}(x)$. Further, *M* denotes a constant not necessarily the same at each occurrence.

The Lipschitz type space [1] is defined as follows:

5 $$ \operatorname{Lip}^{*}_{M}(r):= \biggl\{ f\in C [0,\infty ) : \bigl\vert f(t)-f(x) \bigr\vert \leq M_{f}\frac {|t-x|^{r}}{(t+x)^{r/2}}; x,t>0 \biggr\} $$

for some $M_{f}>0$ and $0< r\leq1$. Several researchers have considered the approximation of functions in this space for different sequences of linear positive operators (cf. [12, 14–16] etc.).

### Theorem 3.1

*Let* $f \in \operatorname{Lip}^{*}_{M}(r)$ *and* $r\in(0,1]$, *then* $\forall x>0$

$$ \bigl\vert L_{n}^{\alpha_{n},\beta_{n}}(f;x)-f(x) \bigr\vert \leq M_{f} \biggl(\frac{\delta_{\alpha _{n},\beta_{n}}(x)}{\sqrt{x}} \biggr)^{r}. $$

### Proof

Since $f \in \operatorname{Lip}^{*}_{M}(r)$, by definition (5)

$$ \bigl\vert f(t)-f(x) \bigr\vert \leq M_{f} \frac{ \vert t-x \vert ^{r}}{(t+x)^{r/2}}. $$

Operating by $L_{n}^{\alpha_{n},\beta_{n}}$ on the above equation, we get

$$ \bigl\vert L_{n}^{\alpha_{n},\beta_{n}}(f;x)-f(x) \bigr\vert \leq M_{f} L_{n}^{\alpha_{n},\beta_{n}} \biggl(\frac{ \vert t-x \vert ^{r}}{(t+x)^{r/2}};x \biggr). $$

Now, applying Hölder’s inequality with $p=2/r$, $q= 2/(2-r)$ and using Lemma 2.1, we get

$$\begin{aligned} \bigl\vert L_{n}^{\alpha_{n},\beta_{n}}(f;x)-f(x) \bigr\vert \leq& M_{f} \biggl(L_{n}^{\alpha_{n},\beta _{n}} \biggl( \frac{(t-x)^{2}}{(t+x)};x \biggr) \biggr)^{r/2} \bigl(L_{n}^{\alpha_{n},\beta_{n}}(1;x) \bigr)^{(2-r)/2} \\ \leq& \frac{M_{f}}{x^{r/2}} \bigl(L_{n}^{\alpha_{n},\beta_{n}} \bigl((t-x)^{2};x \bigr) \bigr)^{r/2}, \end{aligned}$$

which gives the required result. □

Let $\tilde{C}_{B}[0,\infty)$ denote the space of all real-valued bounded and uniformly continuous functions *f* on $[0,\infty)$, endowed with the norm $\| f\|_{\infty} = \sup_{x\in[0,\infty)} |f(x)|$. Further, we obtain a local direct estimate for the operators (1) using the Lipschitz maximal function of order *r* introduced by Lenze [17]

6 $$ \tilde{\omega}_{r}(f;x)=\sup_{t\neq x, t\in[0,\infty)} \frac {|f(t)-f(x)|}{|t-x|^{r}}, $$

where $x\in[0,\infty)$ and $r\in(0,1]$. For contributions of researchers on approximation of functions in this space, we refer to [12, 14, 15, 18].

### Theorem 3.2

*Let* $f\in\tilde{C}_{B}[0,\infty)$ *and* $r\in(0,1]$, *then* $\forall x\in [0,\infty)$

$$\bigl\vert L_{n}^{\alpha_{n},\beta_{n}}(f;x)-f(x) \bigr\vert \leq\tilde{ \omega}_{r}(f;x) \bigl(\delta _{\alpha_{n},\beta_{n}}(x)\bigr)^{r}. $$

### Proof

By equation (6),

$$\bigl\vert f(t)-f(x) \bigr\vert \leq\tilde{\omega}_{r}(f;x){|t-x|^{r}}. $$

Applying the operator $L_{n}^{\alpha_{n},\beta_{n}}$ on both sides of the above equation, and then using Lemma 2.1 and Hölder’s inequality with $p=2/r$, $q= 2/(2-r)$, we get

$$\begin{aligned} \bigl\vert L_{n}^{\alpha_{n},\beta_{n}}(f;x)-f(x) \bigr\vert \leq& \tilde{\omega }_{r}(f;x)L_{n}^{\alpha_{n},\beta_{n}}\bigl( \vert t-x \vert ^{r};x\bigr) \\ \leq& \tilde{\omega}_{r}(f;x) \bigl(L_{n}^{\alpha_{n},\beta _{n}} \bigl((t-x)^{2};x\bigr) \bigr)^{r/2} \bigl(L_{n}^{\alpha_{n},\beta_{n}}(1;x) \bigr)^{(2-r)/2} \\ =& \tilde{\omega}_{r}(f;x) \bigl(\delta_{\alpha_{n},\beta_{n}}(x) \bigr)^{r}. \end{aligned}$$

Thus, we get the desired result. □

For $f\in\tilde{C}_{B}[0,\infty)$ and $\delta>0$, the first order modulus of continuity is defined as

7 $$ \omega(f;\delta)= \sup_{0< |h|\leq\delta} \sup _{x,x+h\in[0,\infty)} \bigl\vert f(x+h)-f(x) \bigr\vert , $$

and Peetre’s K-functional is given by

$$ K(f;\delta) = \inf_{g\in\tilde{C}^{2}_{B}[0,\infty)} \bigl\{ \Vert f-g \Vert _{\infty} + \delta\|g\|_{\tilde{C}^{2}_{B}}\bigr\} , $$

where $\tilde{C}^{2}_{B}[0,\infty)= \{ g\in\tilde{C}_{B}[0,\infty): g',g''\in\tilde{C}_{B}[0,\infty) \}$ with norm $\|g\|_{\tilde{C}^{2}_{B}}= \| g\|_{\infty}+ \|g'\|_{\infty}+ \|g''\|_{\infty}$. Also

$$ K(f;\delta)\leq M \bigl(\omega_{2}(f;\sqrt{\delta})+ \min\{1,\delta\} \|f\| _{\infty}\bigr) $$

holds for all $\delta>0$ and where $\omega_{2}$ is the second order modulus of smoothness of $f\in\tilde{C}_{B}[0,\infty)$, which is defined as follows:

$$ \omega_{2}(f;\delta)= \sup_{0< |h|\leq\delta} \sup _{x,x+2h\in[0,\infty)} \bigl\vert f(x+2h)-2f(x+h)+f(x) \bigr\vert . $$

### Theorem 3.3

*If* $f\in\tilde{C}_{B}[0,\infty)$, *then*

$$ \bigl\vert L_{n}^{\alpha_{n},\beta_{n}}(f;x)-f(x) \bigr\vert \leq 4K \bigl(f;k_{\alpha_{n},\beta_{n}}(x)\bigr) + \omega\bigl(f,\gamma_{\alpha_{n},\beta_{n}}(x)\bigr), $$

*where* $k_{\alpha_{n},\beta_{n}}(x) = (\delta^{2}_{\alpha_{n},\beta_{n}}(x) + \gamma^{2}_{\alpha_{n},\beta_{n}}(x) )/4$. *Furthermore*,

$$\begin{aligned} \bigl\vert L_{n}^{\alpha_{n},\beta_{n}}(f;x)-f(x) \bigr\vert \leq& M \bigl(\omega_{2} \bigl(f;\sqrt {k_{\alpha_{n},\beta_{n}}(x)} \bigr) + \min\bigl\{ 1,k_{\alpha_{n},\beta_{n}}(x)\bigr\} \|f\|_{\infty} \bigr) \\ &{} + \omega \bigl(f, \bigl\vert \gamma_{\alpha_{n},\beta_{n}}(x) \bigr\vert \bigr). \end{aligned}$$

### Proof

For $f\in\tilde{C}_{B}[0,\infty)$, we define the auxiliary operator as follows:

8 $$ \tilde{L}_{n}^{\alpha_{n},\beta_{n}}(f;x) = L_{n}^{\alpha_{n},\beta_{n}}(f;x) - f \biggl( \frac{\alpha_{n}}{\beta_{n}} \frac{B'(\alpha_{n} x)}{B(\alpha_{n} x)} x + \frac{1}{\beta_{n}} \frac{2A'(1)+A(1)}{2A(1)} \biggr) + f(x). $$

After taking the modulus of both sides and applying Lemma 2.1, we obtain

9 $$\begin{aligned} \bigl\vert \tilde{L}_{n}^{\alpha_{n},\beta_{n}}(f;x) \bigr\vert \leq& \bigl\vert L_{n}^{\alpha_{n},\beta _{n}}(f;x) \bigr\vert + \biggl\vert f \biggl( \frac{\alpha_{n}}{\beta_{n}} \frac{B'(\alpha_{n} x)}{B(\alpha_{n} x)} x + \frac{1}{\beta_{n}} \frac{2A'(1)+A(1)}{2A(1)} \biggr) \biggr\vert \\ &{} + \bigl\vert f(x) \bigr\vert \\ \leq& \|f\|_{\infty} \bigl\vert L_{n}^{\alpha_{n},\beta_{n}}(1;x) \bigr\vert + \|f\|_{\infty} +\|f\|_{\infty} \\ =& 3\|f\|_{\infty}. \end{aligned}$$

Now, by Lemma 2.1, we get

$$ \tilde{L}_{n}^{\alpha_{n},\beta_{n}}(t;x) = x, $$

and therefore

10 $$ \tilde{L}_{n}^{\alpha_{n},\beta_{n}}(t-x;x) = 0. $$

Let $g\in\tilde{C}_{B}^{2}[0,\infty)$, then using Taylor’s theorem we can write

$$ g(t)= g(x)+(t-x)g'(x)+ \int_{x}^{t} (t-u)g''(u) \,du. $$

Operating $\tilde{L}_{n}^{\alpha_{n},\beta_{n}}$ on the above equation and using (10), we get

$$\begin{aligned}& \tilde{L}_{n}^{\alpha_{n},\beta_{n}}(g;x)-g(x) \\& \quad = \tilde{L}_{n}^{\alpha_{n},\beta_{n}} \biggl( \int_{x}^{t} (t-u) g''(u) \,du; x \biggr) \\& \quad = L_{n}^{\alpha_{n},\beta_{n}} \biggl( \int_{x}^{t} (t-u) g''(u) \,du; x \biggr) \\& \qquad {} - \int_{x}^{ \frac{\alpha_{n}}{\beta_{n}} \frac{B'(\alpha_{n} x)}{B(\alpha_{n} x)} x + \frac{1}{\beta_{n}} \frac{2A'(1)+A(1)}{2A(1)} } \biggl( \frac{\alpha_{n}}{\beta_{n}} \frac{B'(\alpha_{n} x)}{B(\alpha_{n} x)} x + \frac {1}{\beta_{n}} \frac{2A'(1)+A(1)}{2A(1)} -u \biggr) g''(u) \,du. \end{aligned}$$

Now taking the modulus on both sides, we obtain

$$\begin{aligned}& \bigl\vert \tilde{L}_{n}^{\alpha_{n},\beta_{n}}(g;x)-g(x) \bigr\vert \\& \quad \leq \biggl\vert L_{n}^{\alpha_{n},\beta_{n}} \biggl( \int_{x}^{t} (t-u) g''(u) \,du; x \biggr) \biggr\vert \\& \qquad {}+ \biggl\vert \int_{x}^{ \frac{\alpha_{n}}{\beta_{n}} \frac{B'(\alpha_{n} x)}{B(\alpha_{n} x)} x + \frac{1}{\beta_{n}} \frac{2A'(1)+A(1)}{2A(1)} } \biggl( \frac{\alpha_{n}}{\beta_{n}} \frac{B'(\alpha_{n} x)}{B(\alpha_{n} x)} x + \frac {1}{\beta_{n}} \frac{2A'(1)+A(1)}{2A(1)} -u \biggr) g''(u) \,du \biggr\vert \\& \quad \leq L_{n}^{\alpha_{n},\beta_{n}} \biggl( \biggl\vert \int_{x}^{t} \bigl\vert (t-u) \bigr\vert \bigl\vert g''(u) \bigr\vert \,du \biggr\vert ; x \biggr) \\& \qquad {}+ \biggl\vert \biggl( \int_{x}^{ \frac{\alpha_{n}}{\beta_{n}} \frac{B'(\alpha_{n} x)}{B(\alpha_{n} x)} x + \frac{1}{\beta_{n}} \frac{2A'(1)+A(1)}{2A(1)} } \biggl\vert \frac{\alpha_{n}}{\beta_{n}} \frac{B'(\alpha_{n} x)}{B(\alpha_{n} x)} x + \frac {1}{\beta_{n}} \frac{2A'(1)+A(1)}{2A(1)} -u \biggr\vert \bigl\vert g''(u) \bigr\vert \,du \biggr) \biggr\vert \\& \quad \leq \bigl\Vert g'' \bigr\Vert _{\infty} \biggl\{ L_{n}^{\alpha_{n},\beta_{n}} \biggl( \biggl\vert \int _{x}^{t} \bigl\vert (t-u) \bigr\vert \,du \biggr\vert ; x \biggr) \\& \qquad {}+ \biggl\vert \biggl( \int_{x}^{ \frac{\alpha_{n}}{\beta_{n}} \frac {B'(\alpha_{n} x)}{B(\alpha_{n} x)} x + \frac{1}{\beta_{n}} \frac {2A'(1)+A(1)}{2A(1)} } \biggl\vert \frac{\alpha_{n}}{\beta_{n}} \frac{B'(\alpha_{n} x)}{B(\alpha_{n} x)} x + \frac {1}{\beta_{n}} \frac{2A'(1)+A(1)}{2A(1)} -u \biggr\vert \,du \biggr) \biggr\vert \biggr\} . \end{aligned}$$

Therefore, by using $\|g\|_{\tilde{C}^{2}_{B}}= \|g\|_{\infty}+ \|g'\| _{\infty}+ \|g''\|_{\infty}$,

11 $$\begin{aligned}& \bigl\vert \tilde{L}_{n}^{\alpha_{n},\beta_{n}}(g;x)-g(x) \bigr\vert \\& \quad \leq \|g\|_{\tilde{C}_{B}^{2}} \biggl\{ L_{n}^{\alpha_{n},\beta_{n}} \bigl( (t-x)^{2}; x \bigr) + \biggl( \frac{\alpha_{n}}{\beta_{n}} \frac{B'(\alpha_{n} x)}{B(\alpha_{n} x)} x + \frac{1}{\beta_{n}} \frac{2A'(1)+A(1)}{2A(1)} -x \biggr)^{2} \biggr\} \\& \quad \leq \|g\|_{\tilde{C}_{B}^{2}} \bigl\{ L_{n}^{\alpha_{n},\beta_{n}} \bigl( (t-x)^{2}; x \bigr) + \bigl( L_{n}^{\alpha_{n},\beta_{n}}( t-x; x) \bigr)^{2} \bigr\} \\& \quad = \|g\|_{\tilde{C}_{B}^{2}} \bigl\{ \delta^{2}_{\alpha_{n},\beta_{n}}(x) + \gamma ^{2}_{\alpha_{n},\beta_{n}}(x) \bigr\} . \end{aligned}$$

Now, using the definition of auxiliary operators (8), we have

$$\begin{aligned}& \bigl\vert L_{n}^{\alpha_{n},\beta_{n}}(f;x)-f(x) \bigr\vert \\& \quad \leq \biggl\vert \tilde{L}_{n}^{\alpha_{n},\beta_{n}}(f;x) - f(x) + f \biggl( \frac{\alpha_{n}}{\beta_{n}} \frac{B'(\alpha_{n} x)}{B(\alpha_{n} x)} x + \frac {1}{\beta_{n}} \frac{2A'(1)+A(1)}{2A(1)} \biggr) - f(x) \biggr\vert \\& \quad \leq \bigl\vert \tilde{L}_{n}^{\alpha_{n},\beta_{n}}(f-g;x) \bigr\vert + \bigl\vert \tilde{L}_{n}^{\alpha _{n},\beta_{n}}(g;x)-g(x) \bigr\vert + \bigl\vert g(x)-f(x) \bigr\vert \\& \qquad {} + \biggl\vert f \biggl( \frac{\alpha_{n}}{\beta_{n}} \frac{B'(\alpha_{n} x)}{B(\alpha_{n} x)} x + \frac{1}{\beta_{n}} \frac{2A'(1)+A(1)}{2A(1)} \biggr) - f(x) \biggr\vert . \end{aligned}$$

Combining (7), (9) and (11) with the above equation, we get

$$\begin{aligned}& \bigl\vert L_{n}^{\alpha_{n},\beta_{n}}(f;x)-f(x) \bigr\vert \\& \quad \leq 4\|f-g\|_{\infty} + \|g\|_{\tilde{C}_{B}^{2}} \bigl\{ \delta^{2}_{\alpha _{n},\beta_{n}}(x) + \gamma^{2}_{\alpha_{n},\beta_{n}}(x) \bigr\} + \omega\bigl(f, \bigl\vert \gamma_{\alpha_{n},\beta_{n}}(x) \bigr\vert \bigr) \\& \quad \leq 4\|f-g\|_{\infty} + \|g\|_{\tilde{C}_{B}^{2}} 4 k_{\alpha_{n},\beta _{n}}(x) + \omega\bigl(f, \bigl\vert \gamma_{\alpha_{n},\beta_{n}}(x) \bigr\vert \bigr), \end{aligned}$$

and, after taking the infimum on the right-hand side over all $g\in \tilde{C}_{B}^{2}$, we obtain

$$\begin{aligned}& \bigl\vert L_{n}^{\alpha_{n},\beta_{n}}(f;x)-f(x) \bigr\vert \\& \quad \leq 4K \bigl(f;k_{\alpha_{n},\beta _{n}}(x) \bigr) + \omega \bigl(f, \bigl\vert \gamma_{\alpha_{n},\beta_{n}}(x) \bigr\vert \bigr) \\& \quad \leq M \bigl(\omega_{2} \bigl(f;\sqrt{k_{\alpha_{n},\beta_{n}}(x)} \bigr)+ \min\bigl\{ 1,k_{\alpha_{n},\beta_{n}}(x)\bigr\} \|f\|_{\infty} \bigr) \\& \qquad {} + \omega\bigl(f, \bigl\vert \gamma_{\alpha_{n},\beta_{n}}(x) \bigr\vert \bigr). \end{aligned}$$

This completes the proof. □

### Theorem 3.4

*Let* $f\in\tilde{C}_{B}^{1}[0,\infty)$, *then* $\forall x\geq0$ *and* $\delta>0$,

$$ \bigl\vert L_{n}^{\alpha_{n},\beta_{n}}(f;x)-f(x) \bigr\vert \leq \biggl\{ \bigl\vert f'(x) \bigr\vert + 2\omega \biggl(f', \frac{\delta_{\alpha_{n},\beta_{n}}(x)}{2} \biggr) \biggr\} \delta_{\alpha _{n},\beta_{n}}(x). $$

### Proof

Since $f\in\tilde{C}_{B}^{1}[0,\infty)$, we may write

$$ f(t)-f(x) = f'(x) (t-x) + \int_{x}^{t} \bigl(f'(u)-f'(x) \bigr) \,du. $$

Now, using the well-known property of modulus of continuity, for $\delta >0$ and $f\in\tilde{C}_{B}^{1}[0,\infty)$, we get

$$ \bigl\vert \bigl(f'(u)-f'(x)\bigr) \bigr\vert \leq\omega\bigl(f',\delta\bigr) \biggl( \frac{|u-x|}{\delta }+1 \biggr), $$

hence

$$ \biggl\vert \int_{x}^{t} \bigl(f'(u)-f'(x) \bigr)\,du \biggr\vert \leq\omega\bigl(f',\delta \bigr) \biggl( \frac{(t-x)^{2}}{2\delta}+|t-x| \biggr). $$

Therefore,

$$\begin{aligned} \bigl\vert L_{n}^{\alpha_{n},\beta_{n}}(f;x)-f(x) \bigr\vert \leq& \bigl\vert f'(x) \bigr\vert L_{n}^{\alpha_{n},\beta _{n}}\bigl( \vert t-x \vert ;x\bigr) \\ &{} + \omega\bigl(f',\delta\bigr) \biggl( \frac{1}{2\delta} L_{n}^{\alpha_{n},\beta _{n}}\bigl((t-x)^{2};x\bigr)+ L_{n}^{\alpha_{n},\beta_{n}}\bigl( \vert t-x \vert ;x\bigr) \biggr). \end{aligned}$$

After applying the Cauchy-Schwarz inequality, we have

$$\begin{aligned} \begin{aligned} \bigl\vert L_{n}^{\alpha_{n},\beta_{n}}(f;x)-f(x) \bigr\vert \leq{}& \bigl( \bigl\vert f'(x) \bigr\vert + \omega \bigl(f',\delta\bigr) \bigr) \sqrt{L_{n}^{\alpha_{n},\beta_{n}} \bigl((t-x)^{2};x\bigr)} \sqrt{L_{n}^{\alpha_{n},\beta_{n}}(1;x)} \\ &{} + \omega\bigl(f',\delta\bigr) \biggl( \frac{1}{2\delta} L_{n}^{\alpha_{n},\beta _{n}}\bigl((t-x)^{2};x\bigr) \biggr) \\ ={}& \bigl( \bigl\vert f'(x) \bigr\vert + \omega \bigl(f',\delta\bigr) \bigr) \delta_{\alpha_{n},\beta _{n}}(x) + \omega \bigl(f',\delta\bigr) \biggl( \frac{\delta^{2}_{\alpha_{n},\beta _{n}}(x)}{2\delta} \biggr). \end{aligned} \end{aligned}$$

Choosing $\delta= \frac{1}{2}\delta_{\alpha_{n},\beta_{n}}(x)$, we get our result. □

Let $\phi(x)=\sqrt{x}$. For $f\in\tilde{C}_{B}[0,\infty)$, the Ditzian-Totik modulus of smoothness of first order is given by

$$ \omega_{\phi}(f;\delta)= \sup_{0< h\leq\delta} \biggl\{ \biggl\vert f \biggl(x+\frac{h \phi(x)}{2} \biggr) - f \biggl(x-\frac{h \phi(x)}{2} \biggr) \biggr\vert ; x\pm \frac{h\phi(x)}{2}\in [0,\infty ) \biggr\} , $$

and appropriate Peetre’s K-functional is defined by

$$K_{\phi}(f;\delta)= \inf_{g\in W_{\phi}[0,\infty)} \bigl\{ \Vert f-g \Vert _{\infty} + \delta \bigl\Vert \phi g' \bigr\Vert _{\infty} \bigr\} , \quad \delta>0, $$

where $W_{\phi}[0,\infty) := \{ g: g\in{AC}_{\mathrm{loc}}[0,\infty), \|\phi g' \|_{\infty} < \infty\}$ and $g\in{AC}_{\mathrm{loc}}[0,\infty)$ means that *g* is differentiable and absolutely continuous on every compact subset $[a,b]$ of $[0,\infty)$. It is known from [19] that there exists a constant $M>0$ such that

12 $$ M^{-1} \omega_{\phi}(f;\delta) \leq K_{\phi}(f;\delta)\leq M \omega _{\phi}(f;\delta). $$

Now, we find the order of approximation of the operator (1) by means of the Ditzian-Totik modulus of smoothness.

### Theorem 3.5

*For any* $f\in\tilde{C}_{B}[0,\infty)$ *and* $x\in(0,\infty)$,

$$\bigl\vert L_{n}^{\alpha_{n},\beta_{n}}(f;x)-f(x) \bigr\vert \leq M \omega_{\phi} \biggl(f; \frac {\delta_{\alpha_{n},\beta_{n}}(x)}{\sqrt{x}} \biggr). $$

### Proof

By Taylor’s theorem, for any $g\in W_{\phi}[0,\infty)$, we get

$$\begin{aligned} g(t) =& g(x) + \int_{x}^{t} g'(u) \,du \\ =& g(x) + \int_{x}^{t} \frac{g'(u) \phi(u)}{\phi(u)} \,du, \end{aligned}$$

therefore,

$$\begin{aligned} \begin{aligned} \bigl\vert g(t)-g(x) \bigr\vert &\leq \bigl\Vert \phi g' \bigr\Vert _{\infty} \biggl\vert \int_{x}^{t} \frac{1}{\phi (u)} \,du \biggr\vert \\ &= \bigl\Vert \phi g' \bigr\Vert _{\infty}\cdot 2 \vert \sqrt{t}-\sqrt{x} \vert \\ &= 2 \bigl\Vert \phi g' \bigr\Vert _{\infty} \frac{|t-x|}{\sqrt{t}+\sqrt{x}}, \end{aligned} \end{aligned}$$

which gives

$$\begin{aligned} \bigl\vert g(t)-g(x) \bigr\vert \leq& 2 \bigl\Vert \phi g' \bigr\Vert _{\infty} \frac{|t-x|}{\sqrt{x}} \\ =& 2 \bigl\Vert \phi g' \bigr\Vert _{\infty} \frac{|t-x|}{\phi(x)}. \end{aligned}$$

For any $g\in W_{\phi}[0,\infty)$, using Lemma 2.1 and the above equation, we get

$$\begin{aligned} \bigl\vert L_{n}^{\alpha_{n},\beta_{n}}(f;x)-f(x) \bigr\vert \leq& \bigl\vert L_{n}^{\alpha_{n},\beta _{n}}(f-g;x) \bigr\vert + \bigl\vert L_{n}^{\alpha_{n},\beta_{n}}(g;x)-g(x) \bigr\vert + \bigl\vert g(x)-f(x) \bigr\vert \\ \leq& 2 \Vert f-g \Vert _{\infty} + \frac{2 \Vert \phi g' \Vert _{\infty}}{\phi(x)} L_{n}^{\alpha_{n},\beta_{n}}\bigl(|t-x|;x\bigr). \end{aligned}$$

After applying the Cauchy-Schwarz inequality, we obtain

$$\begin{aligned} \bigl\vert L_{n}^{\alpha_{n},\beta_{n}}(f;x)-f(x) \bigr\vert \leq& 2 \|f-g\|_{\infty} + \frac {2\|\phi g'\|_{\infty}}{\phi(x)} \bigl[L_{n}^{\alpha_{n},\beta _{n}} \bigl((t-x)^{2};x\bigr)\bigr]^{1/2} \\ =& 2 \|f-g\|_{\infty} + \frac{2\|\phi g'\|_{\infty}}{\phi(x)} \delta _{\alpha_{n},\beta_{n}}(x). \end{aligned}$$

Taking the infimum on the right-hand side over all $g\in W_{\phi }[0,\infty)$, we get

$$\bigl\vert L_{n}^{\alpha_{n},\beta_{n}}(f;x)-f(x) \bigr\vert \leq2K_{\phi} \biggl(f;\frac{\delta _{\alpha_{n},\beta_{n}}(x)}{\sqrt{x}} \biggr), $$

which leads us to the required result with the help of the relation between Peetre’s K-functional and the Ditzian-Totik modulus of smoothness as given in (12). □

## Weighted approximation

For $\gamma>0$, let $C_{\gamma}[0,\infty) := \{ f\in C[0,\infty) : |f(t)|\leq M_{f}(1+t^{\gamma}), \forall t\geq0 \}$ equipped with the norm

13 $$ \|f\|_{\gamma} =\sup_{t \in[0,\infty)} \frac{|f(t)|}{(1+t^{\gamma})}. $$

Further, let $C^{*}_{2}[0,\infty)$ be the subspace of $C_{2}[0,\infty)$ consisting of functions *f* such that $\lim_{t\rightarrow\infty} \frac {f(t)}{(1+t^{2})}$ exists. Such type of spaces is studied by many mathematicians as described in [20–22] etc.

### Theorem 4.1

*For each* $f\in C^{*}_{2}[0,\infty)$ *and* $r>0$,

$$\lim_{n\rightarrow\infty} \sup_{x\in[0,\infty)} \frac{|L_{n}^{\alpha _{n},\beta_{n}}(f;x)-f(x)|}{(1+x^{2})^{1+r}}=0. $$

### Proof

Let $x_{0}>0$ be arbitrary but fixed, then in view of (13) we get

$$\begin{aligned} \begin{aligned} &\sup_{x\in[0,\infty)} \frac{|L_{n}^{\alpha_{n},\beta _{n}}(f;x)-f(x)|}{(1+x^{2})^{1+r}} \\ &\quad \leq \sup_{x\leq x_{0}} \frac{|L_{n}^{\alpha_{n},\beta _{n}}(f;x)-f(x)|}{(1+x^{2})^{1+r}} + \sup _{x>x_{0}} \frac{|L_{n}^{\alpha_{n},\beta _{n}}(f;x)-f(x)|}{(1+x^{2})^{1+r}} \\ &\quad \leq \sup_{x\leq x_{0}} \bigl\{ \bigl\vert L_{n}^{\alpha_{n},\beta_{n}}(f;x)-f(x) \bigr\vert \bigr\} + \sup _{x>x_{0}} \frac{|L_{n}^{\alpha_{n},\beta_{n}}(f;x)|+|f(x)|}{(1+x^{2})^{1+r}}. \end{aligned} \end{aligned}$$

Since $|f(t)|\leq\|f\|_{2} (1+t^{2})$, hence

14 $$\begin{aligned} \sup_{x\in[0,\infty)} \frac{|L_{n}^{\alpha_{n},\beta _{n}}(f;x)-f(x)|}{(1+x^{2})^{1+r}} \leq& \bigl\Vert L_{n}^{\alpha_{n},\beta _{n}}(f;x)-f(x) \bigr\Vert _{C[0,x_{0}]} \\ &{} + \|f\|_{2} \sup_{x>x_{0}} \frac{|L_{n}^{\alpha_{n},\beta _{n}}(1+t^{2};x)|}{(1+x^{2})^{1+r}} \\ &{} + \sup_{x>x_{0}} \frac{|f(x)|}{(1+x^{2})^{1+r}} \\ =& I_{1}+I_{2}+I_{3}, \quad \mbox{say}. \end{aligned}$$

By the Korovkin theorem, the sequence $\{L_{n}^{\alpha_{n},\beta_{n}}(f;x)\}$ converges uniformly to the function *f* on every closed interval $[0,a]$, as $n\rightarrow\infty$, (cf. [10], p.1491), therefore for a given $\epsilon>0$, $\exists n_{1}\in\mathbb{N}$ such that

15 $$ I_{1}= \bigl\Vert L_{n}^{\alpha_{n},\beta_{n}}(f;x)-f(x) \bigr\Vert _{C[0,x_{0}]} < \frac{\epsilon }{3},\quad \forall n\geq n_{1}. $$

By using Lemma 2.1, we can find $n_{2}\in\mathbb{N}$ such that

$$\begin{aligned}& \bigl\vert L_{n}^{\alpha_{n},\beta_{n}}\bigl(1+t^{2};x\bigr)- \bigl(1+x^{2}\bigr) \bigr\vert < \frac{\epsilon}{3\|f\| _{2}},\quad \forall n \geq n_{2}, \quad \mbox{or}, \\& L_{n}^{\alpha_{n},\beta_{n}}\bigl(1+t^{2};x\bigr) < \bigl(1+x^{2}\bigr)+\frac{\epsilon}{3\|f\| _{2}},\quad \forall n\geq n_{2}. \end{aligned}$$

Hence

16 $$\begin{aligned} I_{2} =& \|f\|_{2} \sup_{x>x_{0}} \frac{|L_{n}^{\alpha_{n},\beta _{n}}(1+t^{2};x)|}{(1+x^{2})^{1+r}} \\ < & \|f\|_{2} \sup_{x>x_{0}} \frac{1}{(1+x^{2})^{1+r}} \biggl( \bigl(1+x^{2}\bigr)+\frac {\epsilon}{3\|f\|_{2}} \biggr),\quad \forall n\geq n_{2} \\ < & \|f\|_{2} \sup_{x>x_{0}} \biggl(\frac{1}{(1+x^{2})^{r}} \biggr) + \frac {\epsilon}{3}, \quad \forall n\geq n_{2} \\ < & \frac{\|f\|_{2}}{(1+x_{0}^{2})^{r}} + \frac{\epsilon}{3},\quad \forall n\geq n_{2}. \end{aligned}$$

Now, using (13), we get

17 $$ I_{3}=\sup_{x>x_{0}} \frac{|f(x)|}{(1+x^{2})^{1+r}} \leq\frac{\|f\|_{2}}{(1+x_{0}^{2})^{r}}. $$

Let $n_{0}=\max\{n_{1},n_{2}\}$, then by (15), (16) and (17)

18 $$ I_{1}+I_{2}+I_{3}< \frac{2\|f\|_{2}}{(1+x_{0}^{2})^{r}} + \frac{2\epsilon}{3},\quad \forall n\geq n_{0}. $$

Choose $x_{0}$ to be so large that

19 $$ \frac{2\|f\|_{2}}{(1+x_{0}^{2})^{r}} < \frac{\epsilon}{3}. $$

Then, combining (14), (18) and (19), we have

$$ \sup_{x\in[0,\infty)} \frac{|L_{n}^{\alpha_{n},\beta _{n}}(f;x)-f(x)|}{(1+x^{2})^{1+r}} < \epsilon, \quad \forall n \geq n_{0}. $$

Hence the proof is done. □

Bojanic [23] studied the rate of convergence for Fourier series of functions of bounded variation. Cheng [24] obtained the rate of convergence of Bernstein polynomials of functions of bounded variation. Later on many researchers have made important contributions in this area of approximation theory (cf. [25–29] etc.). Bojanic and Cheng [30] studied the rate of convergence of Bernstein polynomials for functions with derivatives of bounded variation. Later on, Bojanic and Khan [31] estimated the rate of convergence of some operators of functions with derivatives of bounded variation. Shaw et al. [32] obtained the rates for approximation of functions of bounded variation and for functions with derivatives of bounded variation by positive linear operators. Many mathematicians studied in this direction and their work pertaining to this area is described in the papers [14, 26, 33–38] etc.

Now, we shall obtain the rate of convergence of $L_{n}^{\alpha_{n},\beta _{n}}(f;x)$ for functions having derivatives of bounded variation. Let $\operatorname{DBV}[0,\infty)$ be the space of functions in $C_{2}[0,\infty)$, which have the derivative of bounded variation on every finite subinterval of $[0,\infty)$. Here we show that at the points *x*, where $f'(x+)$ and $f'(x-)$ exist, the operator $L_{n}^{\alpha_{n},\beta_{n}}(f;x)$ converges to the function $f(x)$. A function $f\in \operatorname{DBV}[0,\infty)$ can be represented as

$$ f(x)= \int_{0}^{x} g(t) \,dt + f(0), $$

where *g* denotes a function of bounded variation on every finite subinterval of $[0,\infty)$.

In order to study the convergence of the operators $L_{n}^{\alpha_{n},\beta _{n}}$ for the functions having a derivative of bounded variation, we rewrite the operator (1) as follows:

20 $$ L_{n}^{\alpha_{n},\beta_{n}}(f;x) = \int_{0}^{\infty} W(t,x) f(t) \,dt, $$

where $W(t,x)$ is the kernel given by

$$ W(t,x) = \frac{\beta_{n}}{A(1)B(\alpha_{n} x)} \sum_{k=0}^{\infty} p_{k}(\alpha_{n} x) \chi_{I}(t), $$

$\chi_{I}(t)$ being the characteristic function of $I= [\frac {k}{\beta_{n}},\frac{k+1}{\beta_{n}} ]$.

### Lemma 4.2

*Let for all* $x>0$ *and sufficiently large* *n*

1. $\lambda_{\alpha_{n},\beta_{n}}(t,x) = \int_{0}^{t} W(u,x) \,du \leq \frac{\delta_{\alpha_{n},\beta_{n}}^{2}(x)}{(x-t)^{2}}$, $0\leq t < x$,
2. $1-\lambda_{\alpha_{n},\beta_{n}}(t,x) = \int_{t}^{\infty} W(u,x) \,du \leq\frac{\delta_{\alpha_{n},\beta_{n}}^{2}(x)}{(t-x)^{2}}$, $x\leq t < \infty$.

### Proof

Using Lemma 2.1 and the definition of kernel, we get

$$\begin{aligned} \lambda_{\alpha_{n},\beta_{n}}(t,x) =& \int_{0}^{t} W(u,x) \,du \\ \leq& \int_{0}^{t} \biggl( \frac{x-u}{x-t} \biggr)^{2} W(u,x) \,du \\ \leq& \frac{1}{(x-t)^{2}} \int_{0}^{t} (u-x)^{2} W(u,x) \,du. \end{aligned}$$

Hence

$$\begin{aligned} \lambda_{\alpha_{n},\beta_{n}}(t,x) \leq& \frac{1}{(x-t)^{2}} L_{n}^{\alpha _{n},\beta_{n}} \bigl((u-x)^{2};x\bigr) \\ =& \frac{1}{(x-t)^{2}} \delta_{\alpha_{n},\beta_{n}}^{2}(x). \end{aligned}$$

Similarly, we can prove the other inequality, therefore we omit the details. □

Let $\bigvee_{a}^{b} f$ be the total variation of *f* on $[a,b]$, i.e.,

21 $$ \bigvee_{a}^{b} f = V \bigl(f;[a,b]\bigr)= \sup_{P\in\mathbb{P}} \Biggl(\sum _{i=1}^{n} \bigl\vert f(x_{i})-f(x_{i-1}) \bigr\vert \Biggr),\quad \mbox{bounded variation}, $$

where $\mathbb{P}$ is the set of all partitions $P=\{a=x_{0}, x_{1}, \ldots , x_{n}=b\}$ of $[a,b]$ and it also has the property

$$\bigvee_{a}^{b} f = \bigvee _{a}^{c} f + \bigvee_{c}^{b} f. $$

Let

22 $$ f'_{x}(t) = \textstyle\begin{cases} f'(t)-f'(x-) ,& 0\leq t < x, \\ 0 ,& t=x, \\ f'(t)-f'(x+) ,& x< t < \infty. \end{cases} $$

### Theorem 4.3

*Let* $f\in \operatorname{DBV}[0,\infty)$, $x>0$ *and* *n* *be sufficiently large*, *then*

$$\begin{aligned} \bigl\vert L_{n}^{\alpha_{n},\beta_{n}}(f;x)-f(x) \bigr\vert \leq& \biggl\vert \frac{1}{2} \bigl( f'(x+)+f'(x-) \bigr) \biggr\vert \bigl\vert \gamma_{\alpha_{n},\beta_{n}}(x) \bigr\vert \\ &{} + \frac{\delta^{2}_{\alpha_{n},\beta_{n}}(x)}{x} \sum_{k=1}^{[\sqrt{n}]} \Biggl( \bigvee_{x-x/k}^{x+x/k} f'_{x} \Biggr) + \frac{x}{\sqrt{n}} \Biggl( \bigvee _{x-x/\sqrt{n}}^{x+x/\sqrt{n}} f'_{x} \Biggr) \\ &{} + \frac{\delta^{2}_{\alpha_{n},\beta_{n}}(x)}{x^{2}} \bigl\vert f(2x)-f(x)-x f'(x+) \bigr\vert \\ &{} + \biggl(\frac{M_{f}+|f(x)|}{x^{2}} + 4M_{f} \biggr) \delta^{2}_{\alpha_{n},\beta _{n}}(x) + \bigl\vert f'(x+) \bigr\vert \delta_{\alpha_{n},\beta_{n}}(x) \\ &{} + \biggl\vert \frac{1}{2} \bigl( f'(x+)-f'(x-) \bigr) \biggr\vert \delta _{\alpha_{n},\beta_{n}}(x). \end{aligned}$$

### Proof

By hypothesis (22),

23 $$\begin{aligned} f'(t) =& \frac{1}{2} \bigl( f'(x+)+f'(x-) \bigr) + f'_{x}(t) + \frac {1}{2} \bigl( f'(x+)-f'(x-) \bigr) \operatorname{sgn}(t-x) \\ &{} + \delta_{x}(t) \biggl( f'(t) - \frac{1}{2} \bigl( f'(x+)+f'(x-) \bigr) \biggr), \end{aligned}$$

where

$$\delta_{x}(t) = \textstyle\begin{cases} 1 ,& t=x, \\ 0 ,& t\neq x. \end{cases} $$

Now, using Lemma 2.1, equations (20) and (23), we get

$$\begin{aligned}& L_{n}^{\alpha_{n},\beta_{n}}(f;x)-f(x) \\& \quad = \int_{0}^{\infty} \bigl(f(t)-f(x)\bigr) W(t,x) \,dt \\& \quad = \int_{0}^{\infty} \biggl( \int_{x}^{t} f'(u) \,du \biggr) W(t,x) \,dt \\& \quad = \int_{0}^{\infty} \biggl[ \int_{x}^{t} \biggl\{ \frac{1}{2} \bigl( f'(x+)+f'(x-) \bigr) + f'_{x}(u) + \frac{1}{2} \bigl( f'(x+)-f'(x-) \bigr) \operatorname{sgn}(u-x) \\& \qquad {} + \delta_{x}(u) \biggl( f'(u) - \frac{1}{2} \bigl( f'(x+)+f'(x-) \bigr)\biggr) \biggr\} \,du \biggr] W(t,x) \,dt. \end{aligned}$$

Since $\int_{x}^{t} \delta_{x}(u) \,du = 0$,

24 $$\begin{aligned}& L_{n}^{\alpha_{n},\beta_{n}}(f;x)-f(x) \\& \quad = \frac{1}{2} \bigl( f'(x+)+f'(x-) \bigr) \int_{0}^{\infty} (t-x) W(t,x) \,dt + \int_{0}^{\infty} \biggl( \int_{x}^{t} f'_{x}(u) \,du \biggr) W(t,x) \,dt \\& \qquad {} + \frac{1}{2} \bigl( f'(x+)-f'(x-) \bigr) \int_{0}^{\infty} |t-x| W(t,x) \,dt. \end{aligned}$$

Now, we break the second term on the right-hand side of the above equation as follows:

$$\begin{aligned} \int_{0}^{\infty} \biggl( \int_{x}^{t} f'_{x}(u) \,du \biggr) W(t,x) \,dt =& - \int _{0}^{x} \biggl( \int_{t}^{x} f'_{x}(u) \,du \biggr) W(t,x) \,dt \\ &{} + \int_{x}^{\infty} \biggl( \int_{x}^{t} f'_{x}(u) \,du \biggr) W(t,x) \,dt \\ =& -I_{1}+I_{2}, \end{aligned}$$

where

$$\begin{aligned}& I_{1} = \int_{0}^{x} \biggl( \int_{t}^{x} f'_{x}(u) \,du \biggr) W(t,x) \,dt, \\& I_{2} = \int_{x}^{\infty} \biggl( \int_{x}^{t} f'_{x}(u) \,du \biggr) W(t,x) \,dt. \end{aligned}$$

Taking the modulus on both sides of (24), we get

$$\begin{aligned} \bigl\vert L_{n}^{\alpha_{n},\beta_{n}}(f;x)-f(x) \bigr\vert \leq& \biggl\vert \frac{1}{2} \bigl( f'(x+)+f'(x-) \bigr) \biggr\vert \bigl\vert L_{n}^{\alpha_{n},\beta_{n}}(t-x;x) \bigr\vert + |I_{1}| \\ &{} + |I_{2}| + \biggl\vert \frac{1}{2} \bigl( f'(x+)-f'(x-) \bigr) \biggr\vert L_{n}^{\alpha_{n},\beta_{n}}\bigl( \vert t-x \vert ;x\bigr). \end{aligned}$$

After applying the Cauchy-Schwarz inequality, we get

25 $$\begin{aligned} \bigl\vert L_{n}^{\alpha_{n},\beta_{n}}(f;x)-f(x) \bigr\vert \leq& \biggl\vert \frac{1}{2} \bigl( f'(x+)+f'(x-) \bigr) \biggr\vert \bigl\vert \gamma_{\alpha_{n},\beta_{n}}(x) \bigr\vert + |I_{1}| + |I_{2}| \\ &{} + \biggl\vert \frac{1}{2} \bigl( f'(x+)-f'(x-) \bigr) \biggr\vert \sqrt {L_{n}^{\alpha_{n},\beta_{n}} \bigl((t-x)^{2};x\bigr)} \\ =& \biggl\vert \frac{1}{2} \bigl( f'(x+)+f'(x-) \bigr) \biggr\vert \bigl\vert \gamma _{\alpha_{n},\beta_{n}}(x) \bigr\vert + |I_{1}| + |I_{2}| \\ &{} + \biggl\vert \frac{1}{2} \bigl( f'(x+)-f'(x-) \bigr) \biggr\vert \delta _{\alpha_{n},\beta_{n}}(x). \end{aligned}$$

Now applying Lemma 4.2 and integration by parts, $I_{1}$ can be written as

$$\begin{aligned} I_{1} =& \int_{0}^{x} \biggl( \int_{t}^{x} f'_{x}(u) \,du \biggr) W(t,x) \,dt \\ =& \int_{0}^{x} \biggl( \int_{t}^{x} f'_{x}(u) \,du \biggr) \frac{\partial }{\partial t} \lambda_{\alpha_{n},\beta_{n}}(t,x) \,dt \\ =& \int_{0}^{x} f'_{x}(t) \lambda_{\alpha_{n},\beta_{n}}(t,x) \,dt. \end{aligned}$$

After taking the modulus, we have

$$\begin{aligned} \vert I_{1} \vert \leq& \int_{0}^{x} \bigl\vert f'_{x}(t) \bigr\vert \lambda_{\alpha_{n},\beta_{n}}(t,x) \,dt \\ \leq& \int_{0}^{x-x/\sqrt{n}} \bigl\vert f'_{x}(t) \bigr\vert \lambda_{\alpha_{n},\beta_{n}}(t,x) \,dt + \int_{x-x/\sqrt{n}}^{x} \bigl\vert f'_{x}(t) \bigr\vert \lambda_{\alpha_{n},\beta_{n}}(t,x) \,dt \\ =& K_{1}+K_{2}, \quad \mbox{say}. \end{aligned}$$

Since $f'_{x}(x)=0$, by hypothesis (22),

$$ K_{1} = \int_{0}^{x-x/\sqrt{n}} \bigl\vert f'_{x}(t)-f'_{x}(x) \bigr\vert \lambda_{\alpha_{n},\beta _{n}}(t,x) \,dt. $$

Now, using Lemma 4.2, we get

$$ K_{1} \leq \delta^{2}_{\alpha_{n},\beta_{n}}(x) \int_{0}^{x-x/\sqrt{n}} \bigl\vert f'_{x}(t)-f'_{x}(x) \bigr\vert \frac{dt}{(x-t)^{2}}. $$

By the definition of total variation (21) and taking $t=x-x/u$, we have

$$\begin{aligned} K_{1} \leq& \delta^{2}_{\alpha_{n},\beta_{n}}(x) \int_{0}^{x-x/\sqrt{n}} \Biggl( \bigvee _{t}^{x} f'_{x} \Biggr) \frac{dt}{(x-t)^{2}} \\ =& \delta^{2}_{\alpha_{n},\beta_{n}}(x) \int_{1}^{\sqrt{n}} \Biggl( \bigvee _{x-x/u}^{x} f'_{x} \Biggr) \frac{du}{x}. \end{aligned}$$

Now, after breaking integral into summation,

$$\begin{aligned} K_{1} \leq& \frac{\delta^{2}_{\alpha_{n},\beta_{n}}(x)}{x} \sum_{k=1}^{[\sqrt {n}]} \int_{k}^{k+1} \Biggl( \bigvee _{x-x/u}^{x} f'_{x} \Biggr) \,du \\ \leq& \frac{\delta^{2}_{\alpha_{n},\beta_{n}}(x)}{x} \sum_{k=1}^{[\sqrt{n}]} \Biggl( \bigvee_{x-x/k}^{x} f'_{x} \Biggr) \biggl( \int_{k}^{k+1} \,du \biggr) \\ =& \frac{\delta^{2}_{\alpha_{n},\beta_{n}}(x)}{x} \sum_{k=1}^{[\sqrt{n}]} \Biggl( \bigvee_{x-x/k}^{x} f'_{x} \Biggr). \end{aligned}$$

By Lemma 4.2, $\lambda_{\alpha_{n},\beta_{n}}(t,x)\leq 1$ and using hypothesis (22), we have

$$\begin{aligned} K_{2} =& \int_{x-x/\sqrt{n}}^{x} \bigl\vert f'_{x}(t) \bigr\vert \lambda_{\alpha_{n},\beta_{n}}(t,x) \,dt \\ \leq& \int_{x-x/\sqrt{n}}^{x} \bigl\vert f'_{x}(t)-f'_{x}(x) \bigr\vert \,dt. \end{aligned}$$

Now, by the definition of total variation (21),

$$\begin{aligned} K_{2} \leq& \int_{x-x/\sqrt{n}}^{x} \Biggl( \bigvee _{t}^{x} f'_{x} \Biggr) \,dt \\ \leq& \Biggl( \bigvee_{x-x/\sqrt{n}}^{x} f'_{x} \Biggr) \int_{x-x/\sqrt {n}}^{x} \,dt \\ =& \frac{x}{\sqrt{n}} \Biggl( \bigvee_{x-x/\sqrt{n}}^{x} f'_{x} \Biggr). \end{aligned}$$

Hence,

26 $$ |I_{1}| \leq \frac{\delta^{2}_{\alpha_{n},\beta_{n}}(x)}{x} \sum _{k=1}^{[\sqrt {n}]} \Biggl( \bigvee _{x-x/k}^{x} f'_{x} \Biggr) + \frac{x}{\sqrt{n}} \Biggl( \bigvee_{x-x/\sqrt{n}}^{x} f'_{x} \Biggr). $$

Using Lemma 4.2, we can write

$$\begin{aligned} |I_{2}| =& \biggl\vert \int_{x}^{\infty} \biggl( \int_{x}^{t} f'_{x}(u) \,du \biggr) W(t,x) \,dt \biggr\vert \\ \leq& \biggl\vert \int_{x}^{2x} \biggl( \int_{x}^{t} f'_{x}(u) \,du \biggr) \frac {\partial}{\partial t} \bigl(1-\lambda_{\alpha_{n},\beta_{n}}(t,x)\bigr) \,dt \biggr\vert \\ &{} + \biggl\vert \int_{2x}^{\infty} \biggl( \int_{x}^{t} f'_{x}(u) \,du \biggr) W(t,x) \,dt \biggr\vert . \end{aligned}$$

Now, applying integration by parts and hypothesis (22), we obtain

$$\begin{aligned} |I_{2}| \leq& \biggl\vert \int_{x}^{2x} f'_{x}(u) \,du\cdot \bigl(1-\lambda_{\alpha_{n},\beta _{n}}(2x,x)\bigr) - \int_{x}^{2x} f'_{x}(t) \bigl(1-\lambda_{\alpha_{n},\beta_{n}}(t,x)\bigr) \,dt \biggr\vert \\ &{} + \biggl\vert \int_{2x}^{\infty} \biggl( \int_{x}^{t} \bigl(f'(u)-f'(x+) \bigr) \,du \biggr) W(t,x) \,dt \biggr\vert \\ \leq& \biggl\vert \int_{x}^{2x} f'_{x}(u) \,du \biggr\vert \cdot\frac{\delta^{2}_{\alpha _{n},\beta_{n}}(x)}{(x)^{2}} + \int_{x}^{2x} \bigl\vert f'_{x}(t) \bigr\vert \cdot\bigl(1-\lambda_{\alpha _{n},\beta_{n}}(t,x)\bigr) \,dt \\ &{} + \biggl\vert \int_{2x}^{\infty} \bigl(f(t)-f(x)\bigr) W(t,x) \,dt \biggr\vert + \bigl\vert f'(x+) \bigr\vert \biggl\vert \int_{2x}^{\infty} (t-x) W(t,x) \,dt \biggr\vert \\ =& P_{1}+P_{2}+P_{3}+P_{4},\quad \mbox{say}. \end{aligned}$$

Now, by hypothesis (22),

$$\begin{aligned} P_{1} =& \frac{\delta^{2}_{\alpha_{n},\beta_{n}}(x)}{x^{2}} \biggl\vert \int_{x}^{2x} f'_{x}(u) \,du \biggr\vert \\ \leq& \frac{\delta^{2}_{\alpha_{n},\beta_{n}}(x)}{x^{2}} \biggl\vert \int_{x}^{2x} \bigl(f'(u)-f'(x+) \bigr) \,du \biggr\vert \\ \leq& \frac{\delta^{2}_{\alpha_{n},\beta_{n}}(x)}{x^{2}} \bigl\vert f(2x)-f(x)-x f'(x+) \bigr\vert \end{aligned}$$

and

$$\begin{aligned} P_{2} =& \int_{x}^{2x} \bigl\vert f'_{x}(t) \bigr\vert \cdot\bigl(1-\lambda_{\alpha_{n},\beta_{n}}(t,x)\bigr) \,dt \\ =& \int_{x}^{x+x/\sqrt{n}} \bigl\vert f'_{x}(t) \bigr\vert \cdot\bigl(1-\lambda_{\alpha_{n},\beta _{n}}(t,x)\bigr) \,dt \\ &{} + \int_{x+x\sqrt{n}}^{2x} \bigl\vert f'_{x}(t) \bigr\vert \cdot\bigl(1-\lambda_{\alpha_{n},\beta _{n}}(t,x)\bigr) \,dt \\ =& J_{1}+J_{2},\quad \mbox{say}. \end{aligned}$$

Using Lemma 4.2, $1-\lambda_{\alpha_{n},\beta _{n}}(t,x)\leq1$ and hypothesis (22), we get

$$\begin{aligned} J_{1} =& \int_{x}^{x+x/\sqrt{n}} \bigl\vert f'_{x}(t) \bigr\vert \cdot\bigl(1-\lambda_{\alpha_{n},\beta _{n}}(t,x)\bigr) \,dt \\ \leq& \int_{x}^{x+x/\sqrt{n}} \bigl\vert f'_{x}(t)-f'_{x}(x) \bigr\vert \,dt \\ \leq& \int_{x}^{x+x/\sqrt{n}} \Biggl( \bigvee _{x}^{t} f'_{x} \Biggr) \,dt \\ \leq& \Biggl( \bigvee_{x}^{x+x/\sqrt{n}} f'_{x} \Biggr) \int_{x}^{x+x/\sqrt {n}} \,dt \\ \leq& \frac{x}{\sqrt{n}} \Biggl( \bigvee_{x}^{x+x/\sqrt{n}} f'_{x} \Biggr). \end{aligned}$$

Now, again with the help of Lemma 4.2 and by hypothesis (22), we get

$$\begin{aligned} J_{2} =& \int_{x+x\sqrt{n}}^{2x} \bigl\vert f'_{x}(t) \bigr\vert \cdot\bigl(1-\lambda_{\alpha_{n},\beta _{n}}(t,x)\bigr) \,dt \\ \leq& \delta^{2}_{\alpha_{n},\beta_{n}}(x) \int_{x+x\sqrt{n}}^{2x} \bigl\vert f'_{x}(t)-f'_{x}(x) \bigr\vert \frac{dt}{(t-x)^{2}}. \end{aligned}$$

By using (21) and $t=x+x/u$,

$$\begin{aligned} J_{2} \leq& \delta^{2}_{\alpha_{n},\beta_{n}}(x) \int_{x+x\sqrt{n}}^{2x} \Biggl( \bigvee _{x}^{t} f'_{x} \Biggr) \frac{dt}{(t-x)^{2}} \\ =& \delta^{2}_{\alpha_{n},\beta_{n}}(x) \int_{1}^{\sqrt{n}} \Biggl( \bigvee _{x}^{x+x/u} f'_{x} \Biggr) \frac{du}{x} \\ \leq& \frac{\delta^{2}_{\alpha_{n},\beta_{n}}(x)}{x} \sum_{k=1}^{[\sqrt{n}]} \int_{k}^{k+1} \Biggl( \bigvee _{x}^{x+x/u} f'_{x} \Biggr) \,du \\ \leq& \frac{\delta^{2}_{\alpha_{n},\beta_{n}}(x)}{x} \sum_{k=1}^{[\sqrt{n}]} \Biggl( \bigvee_{x}^{x+x/k} f'_{x} \Biggr) \biggl( \int_{k}^{k+1} du \biggr) \\ =& \frac{\delta^{2}_{\alpha_{n},\beta_{n}}(x)}{x} \sum_{k=1}^{[\sqrt{n}]} \Biggl( \bigvee_{x}^{x+x/k} f'_{x} \Biggr). \end{aligned}$$

Hence,

$$ P_{2} \leq \frac{x}{\sqrt{n}} \Biggl( \bigvee _{x}^{x+x/\sqrt{n}} f'_{x} \Biggr) + \frac{\delta^{2}_{\alpha_{n},\beta_{n}}(x)}{x} \sum_{k=1}^{[\sqrt{n}]} \Biggl( \bigvee_{x}^{x+x/k} f'_{x} \Biggr). $$

Now, using the Cauchy-Schwarz inequality, we estimate $P_{4}$

$$\begin{aligned} \begin{aligned} P_{4} &= \bigl\vert f'(x+) \bigr\vert \biggl\vert \int_{2x}^{\infty} (t-x) W(t,x) \,dt \biggr\vert \\ &\leq \bigl\vert f'(x+) \bigr\vert \int_{2x}^{\infty} \vert t-x \vert W(t,x) \,dt \\ &\leq \bigl\vert f'(x+) \bigr\vert \int_{0}^{\infty} \vert t-x \vert W(t,x) \,dt \\ &= \bigl\vert f'(x+) \bigr\vert \sqrt{L_{n}^{\alpha_{n},\beta_{n}} \bigl((t-x)^{2};x\bigr)} \\ &= \bigl\vert f'(x+) \bigr\vert \delta_{\alpha_{n},\beta_{n}}(x). \end{aligned} \end{aligned}$$

Further, as $t\geq2x$, then $2(t-x)\geq t$ and $t-x\geq x$ gives

$$\begin{aligned} P_{3} =& \biggl\vert \int_{2x}^{\infty} \bigl(f(t)-f(x)\bigr) W(t,x) \,dt \biggr\vert \\ \leq& \int_{2x}^{\infty} \bigl\vert f(t) \bigr\vert W(t,x) \,dt + \int_{2x}^{\infty} \bigl\vert f(x) \bigr\vert W(t,x) \,dt \\ \leq& M_{f} \int_{2x}^{\infty} \bigl(1+t^{2}\bigr) W(t,x) \,dt + \bigl\vert f(x) \bigr\vert \int _{2x}^{\infty} W(t,x) \,dt \\ =& \bigl(M_{f} + \bigl\vert f(x) \bigr\vert \bigr) \int_{2x}^{\infty} W(t,x) \,dt + M_{f} \int_{2x}^{\infty } t^{2} W(t,x) \,dt \\ \leq& \bigl(M_{f} + \bigl\vert f(x) \bigr\vert \bigr) \int_{2x}^{\infty} \frac{(t-x)^{2}}{x^{2}} W(t,x) \,dt \\ &{}+ M_{f} \int_{2x}^{\infty} 4(t-x)^{2} W(t,x) \,dt \\ \leq& \biggl(\frac{M_{f}+ \vert f(x) \vert }{x^{2}} + 4M_{f} \biggr) \int_{2x}^{\infty} (t-x)^{2} W(t,x) \,dt \\ \leq& \biggl(\frac{M_{f}+ \vert f(x) \vert }{x^{2}} + 4M_{f} \biggr) L_{n}^{\alpha_{n},\beta _{n}}\bigl((t-x)^{2};x\bigr) \\ =& \biggl(\frac{M_{f}+ \vert f(x) \vert }{x^{2}} + 4M_{f} \biggr) \delta^{2}_{\alpha_{n},\beta_{n}}(x). \end{aligned}$$

Hence,

27 $$\begin{aligned} \vert I_{2} \vert \leq& \frac{\delta^{2}_{\alpha_{n},\beta_{n}}(x)}{x^{2}} \bigl\vert f(2x)-f(x)-x f'(x+) \bigr\vert \\ &{} + \frac{x}{\sqrt{n}} \Biggl( \bigvee_{x}^{x+x/\sqrt{n}} f'_{x} \Biggr) + \frac{\delta^{2}_{\alpha_{n},\beta_{n}}(x)}{x} \sum _{k=1}^{[\sqrt{n}]} \Biggl( \bigvee _{x}^{x+x/k} f'_{x} \Biggr) \\ &{} + \biggl(\frac{M_{f}+ \vert f(x) \vert }{x^{2}} + 4M_{f} \biggr) \delta^{2}_{\alpha_{n},\beta _{n}}(x) + \bigl\vert f'(x+) \bigr\vert \delta_{\alpha_{n},\beta_{n}}(x). \end{aligned}$$

Now from (25)-(27) we obtain

$$\begin{aligned} \bigl\vert L_{n}^{\alpha_{n},\beta_{n}}(f;x)-f(x) \bigr\vert \leq& \biggl\vert \frac{1}{2} \bigl( f'(x+)+f'(x-) \bigr) \biggr\vert \bigl\vert \gamma_{\alpha_{n},\beta_{n}}(x) \bigr\vert + |I_{1}| + |I_{2}| \\ &{} + \biggl\vert \frac{1}{2} \bigl( f'(x+)-f'(x-) \bigr) \biggr\vert \delta _{\alpha_{n},\beta_{n}}(x) \\ \leq& \biggl\vert \frac{1}{2} \bigl( f'(x+)+f'(x-) \bigr) \biggr\vert \bigl\vert \gamma _{\alpha_{n},\beta_{n}}(x) \bigr\vert \\ &{} + \frac{\delta^{2}_{\alpha_{n},\beta_{n}}(x)}{x} \sum_{k=1}^{[\sqrt{n}]} \Biggl( \bigvee_{x-x/k}^{x} f'_{x} \Biggr) + \frac{x}{\sqrt{n}} \Biggl( \bigvee _{x-x/\sqrt{n}}^{x} f'_{x} \Biggr) \\ &{} + \frac{\delta^{2}_{\alpha_{n},\beta_{n}}(x)}{x^{2}} \bigl\vert f(2x)-f(x)-x f'(x+) \bigr\vert \\ &{} + \frac{x}{\sqrt{n}} \Biggl( \bigvee_{x}^{x+x/\sqrt{n}} f'_{x} \Biggr) + \frac{\delta^{2}_{\alpha_{n},\beta_{n}}(x)}{x} \sum _{k=1}^{[\sqrt{n}]} \Biggl( \bigvee _{x}^{x+x/k} f'_{x} \Biggr) \\ &{} + \biggl(\frac{M_{f}+|f(x)|}{x^{2}} + 4M_{f} \biggr) \delta^{2}_{\alpha _{n},\beta_{n}}(x) + \bigl\vert f'(x+) \bigr\vert \delta_{\alpha_{n},\beta_{n}}(x) \\ &{} + \biggl\vert \frac{1}{2} \bigl( f'(x+)-f'(x-) \bigr) \biggr\vert \delta _{\alpha_{n},\beta_{n}}(x), \end{aligned}$$

which finally gives the required result. □

## Conclusion

We study the order of approximation of the Kantorovich-Szász type operators based on Brenke type polynomials with the aid of Peetre’s K-functional and the Ditzian-Totik modulus of smoothness. The rate of convergence of these operators for functions in a Lipschitz type space and a weighted space is investigated. The degree of approximation of functions whose derivatives coincide a.e. with a function of bounded variation is also discussed.
